# Orthopteran Diversity in Steep Slope Vineyards: The Role of Vineyard Type and Vegetation Management

**DOI:** 10.3390/insects14010083

**Published:** 2023-01-13

**Authors:** Vera Wersebeckmann, Carolin Biegerl, Ilona Leyer, Karsten Mody

**Affiliations:** 1 Department of Applied Ecology, Hochschule Geisenheim University, Von-Lade-Str. 1, 65366 Geisenheim, Germany; 2 Department of Animal Ecology and Tropical Biology, University of Würzburg, Biozentrum am Hubland, 97074 Würzburg, Germany

**Keywords:** abandonment, alternating management, biodiversity conservation, grasshopper, insect conservation, succession, traditional land use, vineyard terrace

## Abstract

**Simple Summary:**

Our study evaluated the effects of vineyard abandonment and shrub encroachment and the value of active vineyard management for Orthoptera diversity. We investigated orthopterans in two different vineyard management types (vertically oriented and terraced vineyards) and in vineyard fallows in the Upper Middle Rhine Valley (UMRV) in Germany and determined the effects of local vegetation structures within vineyards and in the surrounding landscape. On the landscape and local scale, woody structures and vineyard abandonment reduced habitat quality, especially for open-adapted Orthoptera species. Management in vineyard inter-rows provided bare ground and supported heat- and open-adapted species while taller vegetation stands on terrace embankments enhanced Orthoptera species richness. We conclude that maintaining viticulture on steep slopes is significant for the preservation of open and herbaceous habitat structures and the conservation of associated Orthoptera species.

**Abstract:**

The abandonment of traditional agricultural practices and subsequent succession are major threats to many open-adapted species and species-rich ecosystems. Viticulture on steep slopes has recently suffered from strong declines due to insufficient profitability, thus increasing the area of fallow land considerably. Changing cultivation systems from vertically oriented to modern vineyard terraces offers an opportunity to maintain management economically viable and thus reduces further abandonment. Hillside parallel terraces favor mechanization, and their embankments offer large undisturbed areas that could provide valuable habitats. We investigated the effects of vineyard abandonment, different vineyard management types (vertically oriented vs. terraced), and local parameters on Orthoptera diversity in 45 study sites along the Upper Middle Rhine Valley in Germany. Our results show that woody structures and vineyard abandonment reduced Orthoptera diversity at the local and landscape scale due to decreased habitat quality, especially for open-adapted species. In contrast, open inter-rows of actively managed vineyard types supported heat-adapted Caelifera species. On terrace embankments, extensive management and taller vegetation benefited Ensifera species, while short and mulched vegetation in vertically oriented vineyards favored the dominance of one single Caelifera species. Our results highlight the significance of maintaining viticultural management on steep slopes for the preservation of both open-adapted Orthoptera species and the cultural landscape.

## 1. Introduction

Insect populations are declining at unprecedented rates [1,2,3]. The causes are much debated but almost certainly include agricultural intensification, land-use change, and the abandonment of traditional agricultural practices [4,5]. Since the second half of the 20th century in Europe, land-use intensification has been a major driver of the loss of farmland biodiversity [6,7]. However, when intensification is not profitable, fields and meadows, especially on difficult-to-access hilly sites, are abandoned and thereby lose much of their ecological value for species adapted to open habitats by natural afforestation [8,9]. 

**Figure 1:**
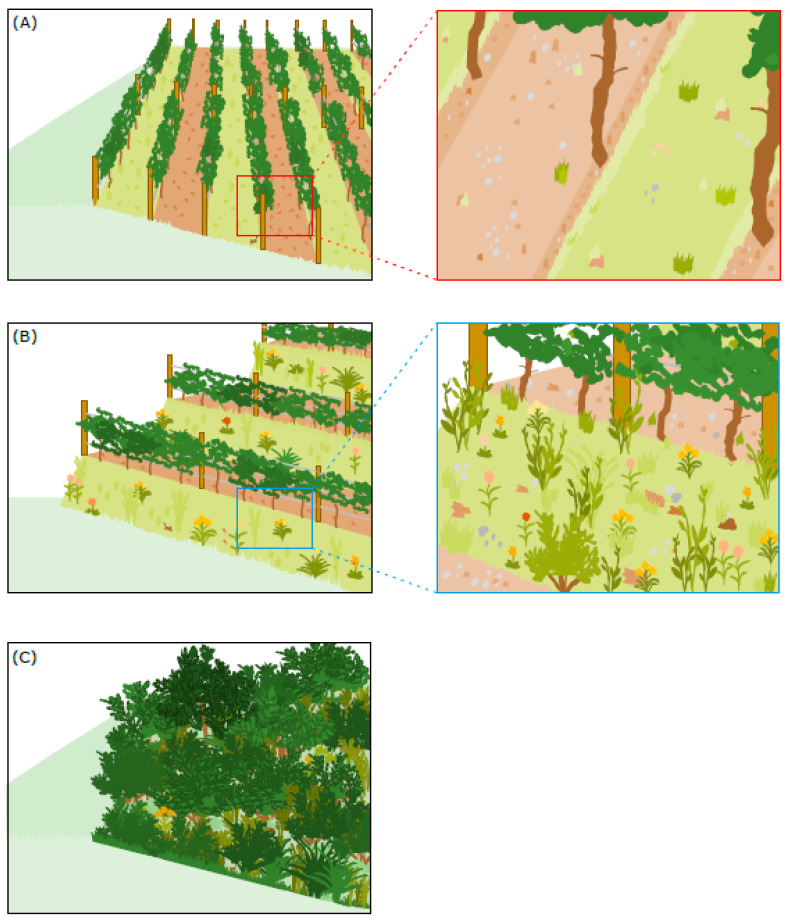
Three vineyard types were studied: (**A**) vertically oriented vineyards that received alternating management with one inter-row being regularly tilled and the other one being permanently covered with vegetation, (**B**) terraced vineyards with regularly tilled inter-rows and extensively managed vegetation on embankments, and (**C**) vineyard fallows that were not managed and abandoned for at least 10 years.

In Germany, viticulture on steep slopes has a long tradition and has shaped entire cultural landscapes such as the Mosel or the Upper Middle Rhine Valley. Traditionally, vines were cultivated on small terraces made of dry-stone walls intermingled with natural elements such as hedges, trees, and clearance cairns [10]. This traditional land-use practice created high structural diversity and provided habitats for many xerothermophilic and nowadays often endangered species, such as the Western green lizard (*Lacerta bilineata*), Red-winged grasshopper (*Oedipoda germanica*), and Scarce swallowtail (*Iphiclides podalirius*) [10,11]. During land consolidation measures in the 1960s, small vineyard terraces were merged into large vineyard sites with downhill-oriented rows (vertically planted vineyards hereafter, see Figure 1A) to increase productivity, mechanization, and the area under cultivation [12]. However, the cultivation of vines on steep slopes still requires substantial manual work, and due to increased labor costs, vertically planted vineyards have become unprofitable [13]. Consequently, steep-slope vineyards have increasingly been abandoned in the past decades (up to 39% in the Middle-Rhine Valley; 1987–2019) [14]. Of the former 2500 hectares in 1909, only about 450 hectares remain in the Middle Rhine Valley today [15]. The progressive expansion of fallow land and ongoing succession threatens flora and fauna adapted to traditional viticultural management and limits the livelihood opportunities of the local population.

One approach to halting viticultural decline on steep slopes is modern vineyard terracing (see Figure 1B). Here, hillside parallel inter-rows favor mechanization, facilitate management and manual work steps, and thus reduce costs considerably [16]. At the same time, terrace embankments offer large uncropped areas between the vines that can provide valuable habitats [17].

Orthopterans are important components of grassland invertebrate assemblages in European agricultural ecosystems [18], particularly due to their significant role as both herbivores and prey for a wide range of taxa, such as birds [19,20,21]. Their high sensitivity and rapid response to environmental changes also make them suitable indicator organisms [18,22]. The impacts of land use change and succession on Orthoptera in European landscapes are well described for open habitats such as grasslands and heathlands [23,24,25], but there is a lack of studies addressing these impacts in vineyards. Moreover, the value of steep slope viticulture for Orthoptera diversity and the effects of various management systems in steep slope viticulture on Orthoptera have not yet been studied. 

This study aims to assess the value of steep slope viticulture for Orthoptera diversity and to evaluate the impacts of ongoing succession as a consequence of vineyard abandonment. In this study, we compared Orthoptera diversity among abandoned vineyards (fallows) and two different vineyard management types (vertically oriented vs. terraced) in a viticultural landscape in Germany and addressed the following research questions: (i) How do Orthoptera species richness, density, and species composition shift across abandoned and managed vineyard types? (ii) How do management-related structures within vineyards affect species richness, density, and species composition? 

## 2. Materials and Methods

### 2.1. Study Area and Sampling Design

**Figure 2:**
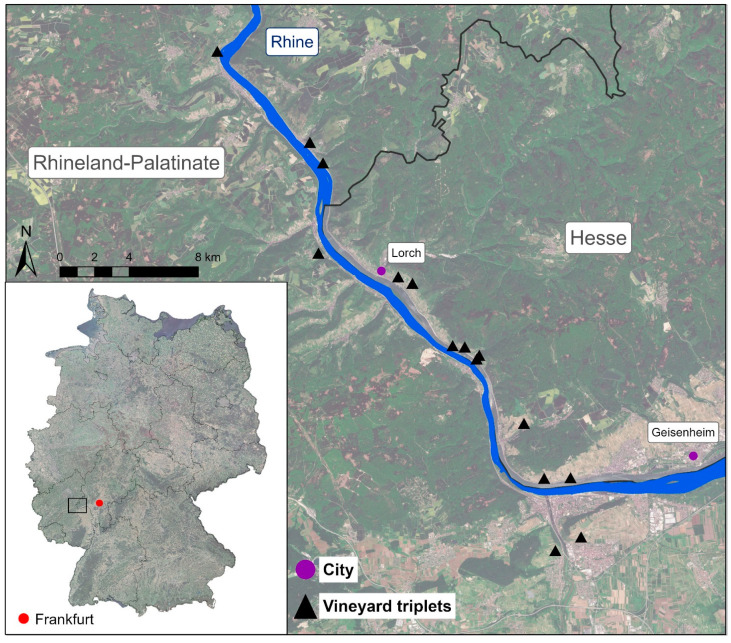
Location of study sites (15 vineyard triplets) in the Upper Middle Rhine Valley. © European Union, contains Copernicus Sentinel-2 data (2021), processed by the German Federal Agency for Cartography and Geodesy (BKG); GeoBasis-DE/BKG 2018.

The study area (75–80 m a.s.l.) is situated in the winegrowing region of the Upper Middle Rhine Valley (UMRV) in Rhineland-Palatinate (50.119139° N, 7.719275° E) and Hesse (50.042342° N, 7.814533° E), Germany (Figure 2). The climate is sub-atlantic, with an annual mean temperature of 12.4 °C and average annual precipitation of 462 mm in the study year (2020) [26]. The soil were clayey loam or loess soils, partially including limestone. Three sites had slate soil. We studied three vineyard types (Figure 1):

(i) *vertically oriented vineyards* received an alternating tillage treatment, i.e., every second inter-row was kept open by regular tillage while the other inter-row was permanently covered with grassy vegetation dominated by *Lolium perenne*. In order to prevent competition for water and nutrients with vines, inter-row vegetation was kept short by regular mulching.
(ii) *terraced vineyards* received a regular tillage treatment in inter-rows, while terrace embankments were permanently covered with grassy and herbaceous vegetation such as *Arrhenaterum elatius*, *Bromus erectus*, *Galium album*, and *Isatis tinctoria*. Embankment vegetation was extensively managed and mulched/mown once in summer.
(iii) *vineyard fallows* were abandoned for at least 10 years and were mostly overgrown with woody vegetation dominated by *Rubus fruticosus* agg., *Rosa canina* agg., *Prunus avium*, and *Crataegus laevigata*.

The vineyard types were arranged in triplets, with each triplet including a vertically oriented vineyard, a terraced vineyard, and a vineyard fallow (Figure 1). We studied a total of 15 triplets (45 sites) belonging to 14 different winegrowers. Therefore, grape variety, age of the vines, and size of the vineyard area differed between sites. In order to ensure similar environmental conditions within triplets, sites were arranged close to each other. The inclination ranged from 17° to 42°.

### 2.2. Orthoptera Sampling

Orthoptera sampling was carried out from late July to mid-August 2020 during sunny and warm weather conditions using suction sampling, according to Mody et al. 2020 [27]. To vacuum a standardized area and to prevent Orthoptera from escaping before sampling, we used a biocenometer (an aluminum frame covered with fine mesh, 1 m × 1 m area, height 0.6 m). 

In managed vineyards (vertically oriented and terraced), we vacuumed a total area of 16 m^2^ per site. To account for structural differences within vineyards, we sampled vegetated and open inter-rows in vertically oriented vineyards and inter-rows and embankments in terraced vineyards (hereafter vineyard compartments) separately. For each of the two vineyard compartments per managed vineyard, we sampled 8 m^2^. Sampling plots (1 m^2^) were spaced at a five-meter distance and split up into two neighboring vineyard compartments of the same type in the center of each site. 

In addition, we also sampled the vine canopy at four randomly picked managed vineyard pairs by constantly vacuuming both sites of the canopy for one minute at two different positions each. Due to great structural differences in vineyard fallows (dense shrubby vegetation) compared to managed vineyards, Orthoptera sampling had to be conducted differently. For vineyard fallows, two sampling locations of about 2 m^2^ were randomly selected in the center and vacuumed for one minute each. 

Orthoptera were directly determined after sampling using the determination keys of Fischer et al. [28]. Species that could not be determined in the field were anesthetized with CO_2_ and frozen until further processing [28,29]. Juvenile Orthoptera that could not be reliably identified were excluded from the analysis. 

### 2.3. Environmental Variables and Landscape Analysis

In parallel with Orthoptera sampling, local vegetation parameters were recorded in two square plots of 1 × 1 m for every vineyard compartment and vineyard fallow. Within each plot, the percentage cover of bare ground, litter, vegetation, and shrubs was visually estimated. In this study, we defined litter as loose dead plant material that frequently occurred in high percentages due to dry and hot weather conditions and vegetation management (mulching). In addition, the average vegetation and shrub height was measured (two random measurements). For data analyses, mean values were calculated.

As landscape context might be a potential determinant of orthopterans [23], the cover of semi-natural habitats (mostly vineyard fallows in our study), forests, and vineyards were quantified in a 150-m radius buffer around the study sites that included the slope range on which vines are cultivated, without including the Rhine. We additionally measured the distance from the center position of each study site to the closest semi-natural habitat and forest. A more detailed description is given in Wersebeckmann et al. 2021 [17].

### 2.4. Data Analysis

For Orthoptera individuals, we calculated the density per m^2^ for each vineyard type and vineyard compartment and assigned Orthoptera to sub-orders (Caelifera and Ensifera). To analyze differences between vineyard types for Orthoptera species richness, density, and local vegetation parameters, (generalized) linear mixed effect models (GLMM) were fitted with vineyard type and vineyard compartment as a fixed effect and ‘vineyard triplet’ (factor with 15 levels) as a random effect to account for the nested design (function “glmmTMB,” package glmmTMB; [30]). Depending on the distribution of the response variable and residuals, models were fitted with the Gaussian family, Poisson family for count data (species richness) or Negative binomial (over-dispersed count data) and Conway-Maxwell (under-dispersed count data) distribution. Post-hoc-tests (contrasts) were performed using the emmeans package [31]. For vineyard compartments, analysis was conducted pairwise within the respective vineyard type (vertically oriented: vegetated vs. open interrow; terraced vineyard: inter-row vs. embankment). For use as a response variable, local vegetation parameters were log-transformed or square-root transformed to reduce skewness (Table 1 and Table 2). To assess the effects of local vineyard and landscape factors on Orthoptera species richness and density and on the density of the two most abundant species within vineyard types and vineyard compartments, we built single GLMMs, due to multicollinearity among predictors, for each combination of the response variable and local and landscape parameters (Y ~ Intercept + predictor + (1|vineyard triplet)). 

To identify species associated with vineyard types and compartments, we performed an indicator species analysis using the IndVal procedure of Dufrêne and Legendre [32]. This analysis combines species abundance with its relative frequency of occurrence within the different vineyard types or compartments (function “multipatt,” package indicspecies; [33]). *p*-values were obtained using a permutation test with 9999 permutations.

To unravel the influence of different vineyard types and compartments on Orthoptera assemblages, indirect ordination methods were applied using the vegan package [34]. To start, we performed a Detrended Correspondence Analysis (DCA) and considered the length of the gradient as a measure of species turnover [35]. Since for vineyard types, the length of gradient was >3 (1st DCA axis: 3.38), the use of a linear-based ordination model was more appropriate, and we performed a Principal Component Analysis (PCA), whereas, for compartments, a DCA (1st DCA axis: 4.02) was more suitable. Local vegetation and landscape parameters that were significantly related to the DCA and PCA axes (*p* < 0.05, based on a permutation test with 9999 permutations) were included post-hoc by projection. Before the analysis, all local vegetation and landscape variables were standardized to zero mean and unit variance (function “scale,” package: vegan). The community data were log10 (x + 1) transformed to reduce the influence of dominant species. Additionally, species with less than two occurrences were excluded from the analysis, thus reducing the number of species from 15 to 7. 

All analyzes were conducted in R version 3.6.3 [36]. Figures and maps were created with ggplot2 [37], cowplot [38], ArcGIS [39], and Inkscape version 1.2.1 [40].

## 3. Results

In total, we sampled 254 Orthoptera individuals and 15 species (Table S1, Supplementary Material). Thereof, seven species (174 individuals, 74.4% of all ind.) belonged to Caelifera and eight species (60 individuals, 25.6% of all ind.) to the Ensifera suborder. *Chorthippus biguttulus* (Linnaeus, 1758) was the most abundant species (112 individuals, 44.0% of all ind.), followed by *Oedipoda caerulescens* (Linnaeus, 1758) with 32 individuals (13.0% of all ind.). Five species were only sampled once (*Chorthippus parallelus* (Zetterstedt, 1821), *Chorthippus dorsatus* (Zetterstedt, 1821), *Nemobius sylvestris* (Bosc, 1792), *Phaneroptera nana* (Fieber, 1853), *Pholidoptera griseoaptera* (De Geer, 1773)). We collected three species of conservation concern, which are listed on the Red List of Orthoptera in Germany [41] (Table S1). In the vineyard canopy, we sampled only two species and three individuals (*L. punctatissima* (1), *O. pellucens* (2)) and therefore did not consider them for further analysis. 

### 3.1. Local Vegetation Parameters

Local vegetation parameters did not differ significantly between managed vineyards (vertically oriented and terraced) but between managed vineyards and fallows (Table 1). Vegetation height and shrub cover were significantly higher, and bare ground and litter cover was significantly lower in fallows compared to managed vineyards. Vegetation cover was significantly higher in vertically oriented vineyards compared to fallows but did not differ from terraced vineyards (Table 1). 

Within managed vineyard types, local vegetation parameters differed significantly between compartments of the respective vineyard type (Table 2). In vertically oriented vineyards, bare ground cover was significantly higher in open inter-rows, while vegetation height, litter, vegetation, and shrub cover were higher in vegetated inter-rows (Table 2). In terraced vineyards, vegetation height, litter, and shrub cover were significantly higher on embankments, while the bare ground cover was significantly higher in inter-rows.

### 3.2. Orthoptera Diversity in Vineyard Types

**Figure 3:**
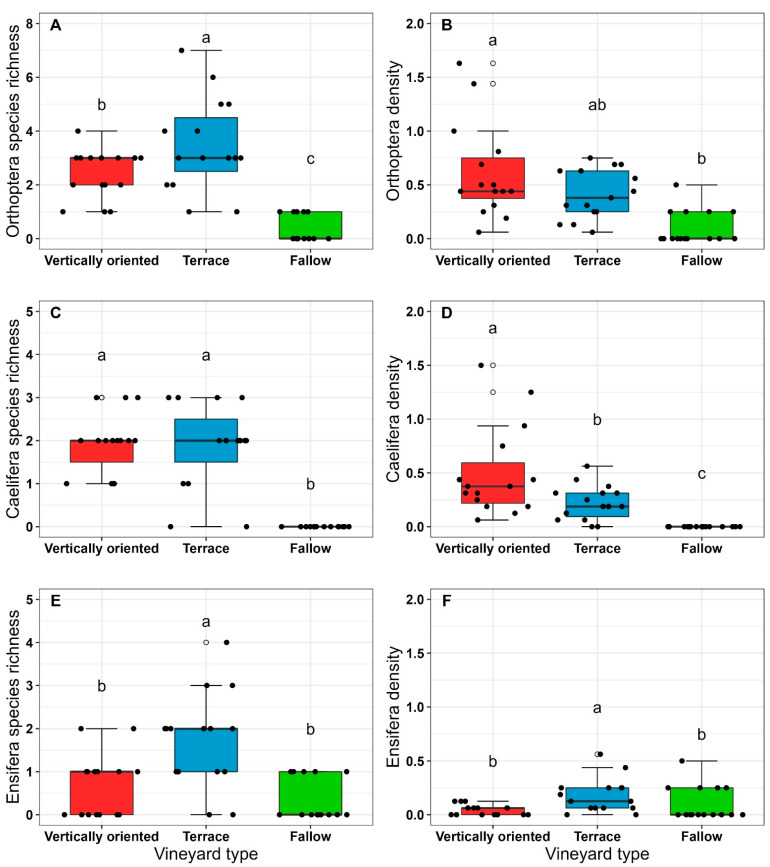
Differences in species richness of Orthoptera (**A**), Caelifera (**C**), Ensifera (**E**), and density of Orthoptera (**B**), Caelifera (**D**), and Ensifera (**F**) between vineyard types were analyzed using (**G** LMMs. Each black-coloured data point represents the number of species or individuals for the respective vineyard type (N = 15). Different letters indicate significant differences between vineyard types (*p* < 0.05).

Orthoptera species richness differed significantly between vineyard types, with terraced vineyards having the highest and fallows having the lowest species richness (Figure 3A). Vertically oriented vineyards had a significantly higher Orthoptera density than vineyard fallows with terraced vineyards in between (Figure 3B). Caelifera species richness was significantly higher in managed vineyard types compared to vineyard fallows (Figure 3C), while Caelifera density was significantly highest in vertically oriented vineyards and lowest in fallows (Figure 3D). Ensifera species richness and density were highest in terraced vineyards compared to vertically oriented vineyards and fallows (Figure 3E,F).

*C. biguttulus* dominated the community in vertically oriented vineyards and was the only indicator species assigned to this vineyard type (Table 3). For terraced vineyards, indicator species analysis revealed four species, three of which belong to the Ensifera-group (*P. albopunctata*, *P. falcata*, *O. pellucens*) and one belonging to the Caelifera-group *(O. caerulescens*) (Table 3). There were no species assigned to vineyard fallows.

### 3.3. Orthoptera Diversity in Vineyard Compartments

**Figure 4:**
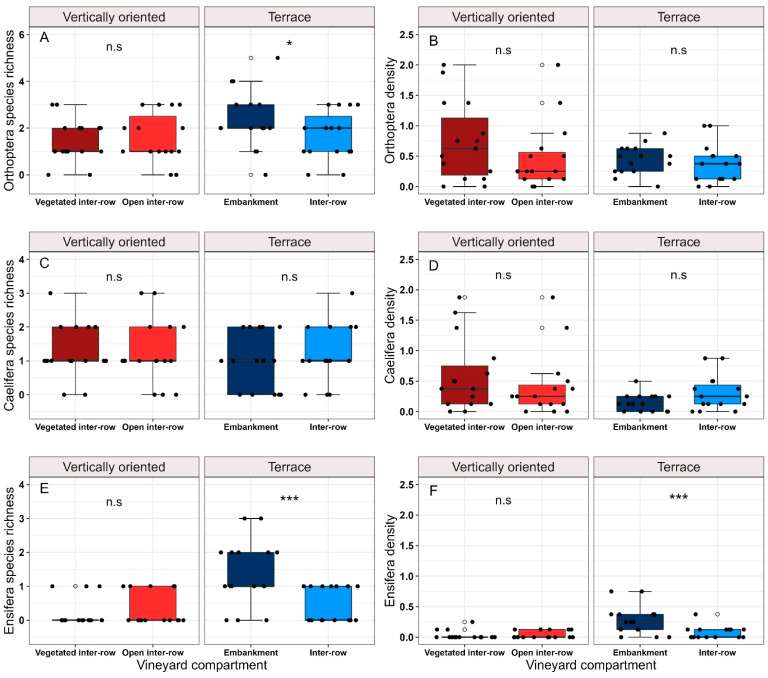
Boxplots show pairwise comparisons of Orthoptera species richness (**A**) and density (**B**), Caelifera species richness (**C**) and density (**D**), and Ensifera species richness (**E**) and density (**F**) between vineyard compartments of vertically oriented and terraced vineyards analyzed using (**G**) LMMs. Each black-coloured data point represents the number of species or individuals for the respective vineyard compartment (N = 15). Asterisks indicate significance levels (*p* < 0.05 = * < 0.001 = ***). Nonsignificant *p*-values are indicated by n.s.

Within vertically oriented vineyards, neither species richness nor density was significantly different between vegetated and open inter-rows (Figure 4A,B). For terraced vineyards, Orthoptera species richness was significantly higher on embankments compared to inter-rows, while Orthoptera density did not differ (Figure 4A,B). There were no differences in Caelifera species richness and density between compartments (Figure 4C,D), while for terraced vineyards, Ensifera species richness and density were significantly higher on embankments compared to inter-rows (Figure 4E,F). Indicator species analysis for compartments revealed two species associated with terrace embankments (Table 4).

### 3.4. Effect of Local and Landscape Parameters on Orthoptera

**Figure 5:**
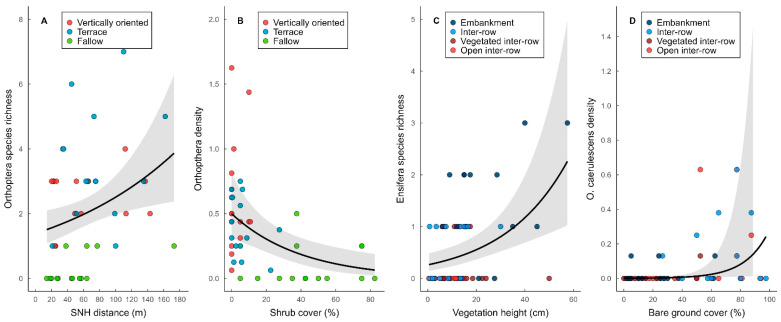
Relationship of Orthoptera species richness with SNH distance (**A**) and Orthoptera density with shrub cover (**B**) analyzed using vineyard types, and Ensifera species richness with vegetation height (**C**) and *Oedipoda caerulescens* density with bare ground cover (**D**) analyzed using vineyard compartments. Grey areas represent SE. See Table 5 (**A**,**B**) and Table S3 (**C**,**D**) for details.

Local vegetation parameters significantly affected Orthoptera, Ensifera, and Caelifera species richness and density and density of the two most abundant species, *C. biguttulus* and *O. caerulescens* in vineyard types and vineyard compartments (Table 5, Tables S2 and S3), whereas these effects were less pronounced for landscape parameters (Table 5, Tables S2 and S3). Among vineyard types, vegetation height and shrub cover negatively affected species richness of Orthoptera, Caelifera, and Ensifera and density of Orthoptera, Caelifera, and *C. biguttulus*, while bare ground cover positively affected Orthoptera and Caelifera species richness (Figure 5B). Vegetation cover had a positive effect on Orthoptera, Caelifera, and *C. biguttulus* density. At the landscape scale, the proximity to semi-natural habitats (SNH) had a positive effect on Orthoptera and Ensifera species richness, while Ensifera density was negatively affected by a cover of SNH but positively affected by vineyard cover in the surrounding landscape (Figure 5A, Table 5 and Table S2). 

In vineyard compartments, vegetation height had a positive effect on Orthoptera and Ensifera species richness (Figure 5C) and Ensifera density, while bare ground cover had a negative effect (Table S3). Ensifera and *C. biguttulus* densities were positively affected by vegetation cover, while *O. carulescens* density was positively affected by a bare ground cover (Figure 5D). Litter cover positively affected Ensifera species richness but had negative effects on *O. caerulescens* density. At the landscape scale, SNH and forest cover positively and vineyard cover negatively affected Caelifera species richness, while *O. caerulescens* was negatively affected by SNH cover in the surrounding landscape (Table S3). 

**Figure 6:**
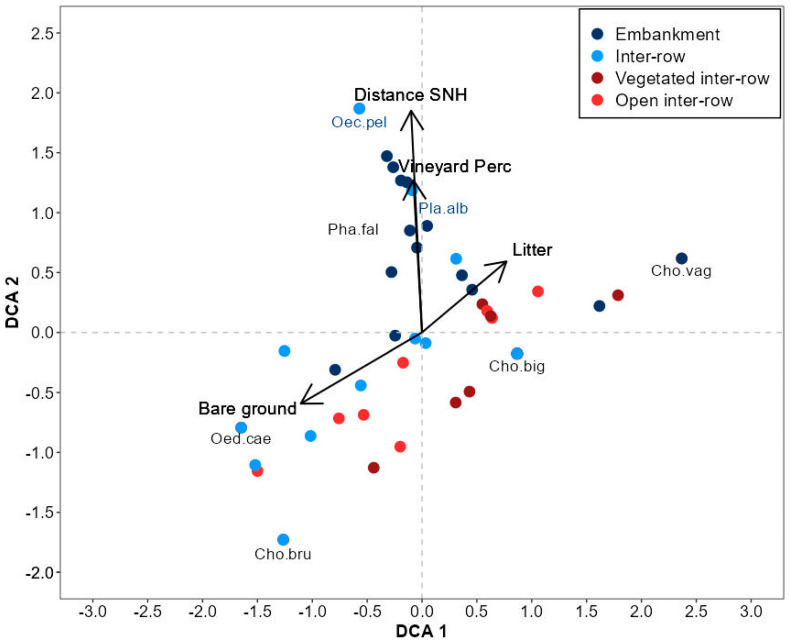
DCA results for Orthoptera species composition of vineyard compartments (length of gradient 1st DCA-axis: 4.02, 2nd DCA-axis: 3.6). Dots show scores for embankment (dark blue), inter-row (light blue), vegetated inter-row (dark red), and open inter-row (light red). Environmental and landscape variables that were significantly related to the DCA axes (*p* < 0.05, 9999 permutations) were included post-hoc by projection. Indicator species of terrace embankments are colored blue. For black-colored species names, no assigned indicator values were available. Species abbreviations: Cho.big: *Chorthippus biguttulus*; Cho.bru: *Chorthippus brunneus*; Cho.vag: *Chorthippus vagans*; Oec.pel: *Oecanthus pellucens*; Oed.cae: *Oedipoda caerulescens*; Pha.alb: *Phaneroptera falcata*; Pla.alb: *Platycleis albopunctata*.

After excluding species with less than two occurrences from the analysis, only two fallow sites remained and revealed no differentiation of Orthoptera communities between vineyard types (Figure S1). In vineyard compartments, the first DCA-axis was mainly correlated with local environmental variables (Table S4). Inter-rows and open inter-rows were associated with higher bare ground cover and *O. caerulescens,* whereas litter cover was more closely associated with embankments (Figure 6). Embankments, as well as the Ensifera species *O. pellucens*, *P. falcata*, and *P. albopunctata,* were positively correlated with the landscape variables distance to SNH and the percentage of surrounding vineyards on the second DCA-axis. 

## 4. Discussion

The orthopteran species we recorded in our study represent approximately 20% of the German Orthoptera fauna [28]. The species richness was similar to other studies investigating Orthoptera in temperate vineyards (10 species in Swiss vineyards, [42]) while Orthoptera density in managed vineyards (density of Ø 0.47/m^2^) appeared to be lower than in other habitats such as grassland (3.5 to 7.4/m^2^) [43,44], field margin strips (0.9 to 3.3/m^2^) [44], or urban roadside vegetation (Ø 1.5, max. 8 ind./m^2^) [27]. 

### 4.1. Effects of Vineyard Abandonment on Orthoptera Diversity

In grasslands and heathlands, Orthoptera associated with open habitats are negatively affected by older successional stages overgrown with woody vegetation resulting in lower species richness [22,25,45]. In our study, we observed a similar pattern as all open-adapted Orthoptera species were missing in vineyard fallows and overall Orthoptera species richness and density were very low in comparison to actively managed vineyards. Orthoptera are cold-blooded organisms that require high ambient temperatures for optimal growth and reproduction [46], which is often interrelated with vegetation structure [47,48]. In fallows, a high and dense shrub cover likely resulted in more shade and hence lower maximum temperatures near the ground. As pointed out by Bieringer and Zulka (2003) [49], shading of the soil surface can be a serious threat for many thermophilous Caelifera species since they require sun-exposed bare ground and high ambient temperatures to complete their life cycles [49,50]. In particular, for critically endangered *O. germanica* that colonizes hot and dry vegetation-free and rocky habitats and is sensitive to denser vegetation, ongoing succession strongly reduces habitat quality [28]. On the contrary, Ensifera species were less affected by the presence of shrubs since they are less sensitive to cooler temperatures and denser vegetation structures for completing their life cycles [49] and are further known to persist better in transitional habitats with ongoing succession [51]. However, the cover of SNHs (foremost vineyard fallows) in the surrounding landscape still negatively affected Ensifera density, while Ensifera species number was positively correlated with an increased distance to SNH at the landscape scale, indicating that this group may be able to persist in these habitats but may still depend on grassy and herbaceous sites at the landscape scale [52]. As forests are not typical habitat for most orthopterans that are typically related to open grasslands, it is not surprising that we found no effects of forest cover in the surrounding landscape. 

Sampling orthopterans in taller vegetation (>50 cm sward height) can be more problematic, and the efficiency of sampling in fallows might be reduced by woody vegetation structures [53]. However, we assume that unfavorable habitat conditions in fallows resulted in low species richness and densities rather than limited sampling efficiency. Accordingly, we assume that vineyard abandonment on steep slopes is a serious threat to Orthoptera diversity and, in particular, to the conservation of open-adapted and heat-loving Caelifera species. Nonetheless, fallows and woody elements in vineyards may have high conservation value for other groups, such as cavity-nesting wild bees [54] or birds [55]. 

### 4.2. Effects of Local Vineyard Management on Orthoptera Diversity

Even though most Orthoptera species are polyphagous and do not depend on specific host plants for their survival, plant community composition is widely considered a determining factor for Orthoptera diversity [56,57]. A diverse plant community provides spatial and structural heterogeneity and hence various habitat niches but also distinct feeding opportunities and shelter against predators [20,48,58]. In our study, distinct vegetation structures were related to different management regimes within vertically oriented and terraced vineyards and determined Orthoptera community composition and contrasting responses of Caelifera and Ensifera species. Orthoptera communities of terrace embankments and vegetated inter-rows were mainly differentiated by distinct vegetation management. While embankment vegetation was mulched only once a year and thus allowed for tall stands of grasses and herbs, the grass-dominated vegetation in vegetated inter-rows was kept short by regular mulching. The Orthoptera community on embankments mainly comprised Ensifera species that prefer a certain vegetation height and are further associated with the presence of shrubs such as *P. falcata* but were still positively correlated with a greater distance to SNHs (foremost vineyard fallows) and cover of vineyards in the surrounding landscape since habitat conditions on vineyard fallows were less suitable (see Section 4.1). The high Orthoptera species richness on embankments was driven by a high Ensifera richness that was positively related to vegetation height. Taller vegetation on embankments is likely to increase shelter for large-bodied Ensifera species that are more prone to vertebrate predators than smaller Caelfiera species [59]. Further, a mosaic of different microhabitats on embankments provided by bare ground patches in addition to taller vegetation met the needs of distinct life stages of Ensifera species such as *P. albopunctata*. This heat- and drought-loving species depends on bare ground patches for oviposition, sparsely vegetated habitats for their nymphs, and taller vegetation for adults [60,61]. In addition, thermophilic *O. pellucens* (Ensifera), typically found on south-exposed embankments and dry shrubbery grasslands, likely profited from high insolation on embankments combined with tall vegetation and a small proportion of shrubs on embankments [28]. 

The relatively high litter cover in our study was the result of withered vegetation due to heat and drought and residuals from mulching in vegetated inter-rows and embankments. Although litter is usually associated with unfavorable habitat conditions for Orthoptera, Gaigher and Samways 2010 [62], found that a moderate litter cover created structural diversity in vineyards and supported different arthropod species. In addition, Bruggisser et al. 2010 [42] suggested that mulching in vineyard inter-rows increased habitat heterogeneity and thus benefited orthopteran diversity in Swiss vineyards. 

The high density of Orthoptera and Caelifera in vertically oriented vineyards was driven by the dominance of *C. biguttulus*, a grass-feeding generalist that colonized a broad range of habitats and seemed to be less sensitive to mulching management and short vegetation in vegetated inter-rows. These results coincide with findings from South African vineyards where Orthoptera diversity was low while Orthoptera density in inter-rows (comparable to vertically oriented vineyards) was high and mostly attributed to the dominance of one single species [63]. Caelifera species richness was similar between terraced vineyards and vertically oriented vineyards, which can be related to the high availability of open and bare ground in both open inter-rows and inter-rows of terraces. For most thermophilic Caelifera species, high temperatures and bare ground patches are essential for completing their life cycle (e.g., oviposition, embryonic development) [45,64]. Most of the individuals observed in open inter-rows belonged to *O. caerulescens,* which is known for its preference for sparsely vegetated, dry, and sun-exposed soils for basking [51]. Nevertheless, *O. caerulescens* depends on a certain amount of herbaceous vegetation for oviposition [28] provided by embankments and vegetated inter-rows. Counterintuitively, when analyzing vineyard compartments, Caelifera species richness showed a positive relationship with SNH and forest cover and a negative relationship with vineyard cover in the surrounding landscape. This effect might be explained by a more frequent occurrence of thermophilic *O. germanica* and *C. vargans* on sites with slate soils that heat up stronger but are even more affected by abandonment and subsequent natural forest recovery as the wine growing at these sites is more effortful and less profitable.

To summarize, Orthoptera diversity in vineyards was supported by habitat heterogeneity created by alternating management within vineyards that provided different microhabitats at a site such as bare ground, short and sparse, and taller vegetation and thus met the diverse requirements many orthopterans have during different life stages [56]. Low-intensity management on embankments provided less disturbed vegetation structures and stands that were particularly beneficial for Ensifera diversity. Nevertheless, largescale mulching, even at low frequencies, causes high mortality rates in orthopterans and other arthropods [62,63] and increases predation rates by vertebrates [65]. These negative effects could be reduced by partial mulching that preserves temporarily uncut vegetation patches and hence provides refuge for orthopterans during and after the cuts [51,66]. Maintaining and improving habitat quality for orthopterans in vineyards is not only important for Orthoptera conservation but offers potential for natural pest control. Recently, in vineyards, omnivorous bush crickets (Tettigonidae) were observed to feed on pupae of the European grapevine moth *Lobesia botrana*, one of the major grapevine pests in Europe [67] and other species such as bush crickets (e.g., *Meconema meridionale*) are known to prey on pests such as leaf miners and chrysomelids [68,69].

## 5. Conclusions

Orthopteran diversity strongly decreased in vineyard fallows, and all open-adapted species disappeared. Therefore, maintaining viticulture on steep slopes to preserve open structures is of great importance both on a local and landscape scale for the conservation of endangered orthopterans, but also for the conservation of habitats for other endangered species such as the Scarce swallowtail (*Iphiclides podalirius*). Faced with the challenges of cost disadvantages in steep slope viticulture, vineyard terracing provides a viable solution to maintaining wine growing economically sustainable and, at the same time, creating valuable habitats within the viticultural system. In particular, the extensive management and low degree of disturbance of the terrace embankments met the distinct habitat requirements that orthopterans have in different life stages by providing both open bare ground areas and vegetative cover.

## Figures and Tables

**Table 1:** Effects of vineyard type on local vegetation parameters (N = 15). Linear mixed effect models were used with vineyard triplet as a random factor. Different letters show significant differences between vineyard types (*p* < 0.05).

	Managed Vineyards		Abandoned Vineyards
Variable	Vertically Oriented	Terrace	Fallow
Vegetation height ^xx^ [cm]	12.43 ^b^ ± 1.80	16.43 ^b^ ± 2.17	69.50 ^a^ ± 9.73
Bare ground cover [%]	36.87 ^a^ ± 5.45	39.98 ^a^ ± 4.71	11.83 ^b^ ± 2.33
Litter cover [%]	37.48 ^a^ ± 4.90	37.99 ^a^ ± 4.83	31.00 ^b^ ± 4.29
Vegetation cover ^xx^ [%]	22.80 ^a^ ± 4.05	16.03 ^ab^ ± 3.35	13.17 ^b^ ± 4.82
Shrub cover ^x^ [%]	2.80 ^b^ ± 1.09	6.00 ^b^ ± 2.13	44.00 ^a^ ± 6.49

^x^ Square-root transformed data were used for testing. ^xx^ Log-transformed data were used for testing.

**Table 2:** Effects of vineyard compartments on local vegetation parameters (N = 15). Comparisons were made pairwise within the respective vineyard type. Different letters show significant differences within the respective vineyard type (*p* < 0.05).

	Vertically Oriented		Terrace	
Variable	Vegetated Inter-Row	Open Inter-Row	Embankment	Inter-Row
Vegetation height [cm]	15.37 ^a^ ± 2.92	9.50 ^b^ ± 1.13	23.87 ^a^ ± 3.82	8.98 ^b^ ± 2.09
Bare ground cover [%]	20.90 ^b^ ± 5.93	52.67 ^a^ ± 7.32	21.80 ^b^ ± 5.05	58.17 ^a^ ± 7.25
Litter cover [%]	46.50 ^a^ ± 6.4	28.13 ^b^ ± 6.07	50.33 ^a^ ± 6.56	25.65 ^b^ ± 5.49
Vegetation cover ^xx^ [%]	27.33 ^a^ ± 5.06	18.10 ^b^ ± 3.64	16.53 ± 4.75	15.52 ± 3.15
Shrub cover ^xx^ [%]	4.50 ^a^ ± 1.73	1.10 ^b^ ± 0.81	11.33 ^a^ ± 4.30	0.67 ^b^ ± 0.52

^xx^ Log-transformed data were used for testing.

**Table 3:** Indicator Orthoptera species for the three vineyard types. Means and standard errors per vineyard type are displayed (N = 15). Species significantly associated with one vineyard type are printed in bold. Respective *p*-values are given (*p* < 0.05).

Species	Vertically Oriented	Terrace	Fallow	*p*
*Platycleis albopunctata*	0.53 ± 0.17	**1.00 ± 0.22**	0.00 ± 0.00	0.0055
*Oecanthus pellucens*	0.13 ± 0.09	**1.33 ± 0.53**	0.20 ± 0.14	0.0056
*Oedipoda caerulescens*	0.93 ± 0.47	**1.20 ± 0.44**	0.00 ± 0.00	0.0451
*Phaneroptera falcata*	0.00 ± 0.00	**0.33 ± 0.16**	0.00 ± 0.00	0.0278
*Chorthippus biguttulus*	**5.80 ± 1.63**	1.67 ± 0.40	0.00 ± 0.00	0.0005

**Table 4:** Indicator Orthoptera species for the four vineyard compartments. Means and standard errors per vineyard compartment are displayed (N = 15). Species significantly associated with one vineyard compartment are printed in bold (*p* < 0.05).

	Vertically Oriented		Terrace		
Species	Vegetated Inter-Row	Open Inter-Row	Embankment	Inter-Row	*p*
*Platycleis albopunctata*	0.27 ± 0.15	0.27 ± 0.12	**0.93 ± 0.23**	0.07 ± 0.07	0.0023
*Oecanthus pellucens*	0.00 ± 0.00	0.07 ± 0.07	**0.87 ± 0.35**	0.40 ± 0.21	0.0103

**Table 5:** Effects of local vineyard and landscape parameters on Orthoptera species richness and density analyzed using vineyard types. The presented estimates and *p*-values are obtained from the single models Y ~ Intercept + Predictor with ‘vineyard triplet’ as a random factor. R^2^ marginal gives explained variation without and R^2^ conditional with the random factor. Significant predictors (*p* < 0.05) are printed in bold; SNH: semi-natural habitat.

Dependent Variable	Predictor	Estimate ± SE	*p*-Value	R^2^_marg_	R^2^_cond_	AICc
Orthoptera species richness	SNH (%)	−0.04 ± 0.12	0.7390	0.03	0.03	175.0
	Vineyard (%)	−0.01 ± 0.13	0.9710	0.01	0.01	175.1
	Forest (%)	0.09 ± 0.13	0.4620	0.02	0.02	174.6
	**SNH distance (m)**	**0.25** **± 0.09**	**0.0086**	**0.14**	**0.01**	**170.3**
	Forest distance (m)	−0.15 ± 0.12	0.1840	0.06	0.01	174.8
	**Vegetation height (cm)**	**−0.85** **± 0.23**	**0.0002**	**0.63**	**0.64**	**153.3**
	**Bare ground cover (%)**	**0.32** **± 0.12**	**0.0081**	**0.18**	**0.18**	**168.2**
	Vegetation cover (%)	0.21 ± 0.12	0.0969	0.07	0.07	172.4
	**Shrub cover (%)**	**−0.85** **± 0.21**	**<0.0001**	**0.64**	**0.64**	**150.6**
	Litter cover (%)	0.07 ± 0.13	0.5820	0.01	0.01	174.8
Orthoptera density	SNH (%)	−0.01 ± 0.05	0.7650	0.02	0.02	43.5
	Vineyard (%)	0.06 ± 0.05	0.2910	0.03	0.03	42.5
	Forest (%)	−0.01 ± 0.05	0.9150	0.01	0.01	43.6
	SNH distance (m)	0.08 ± 0.05	0.1240	0.05	0.00	41.3
	Forest distance (m)	−0.02 ± 0.05	0.6470	0.01	0.01	43.4
	**Vegetation height (cm)**	**−0.18** **± 0.05**	**0.0002**	**0.23**	**0.39**	**32.7**
	Bare ground cover (%)	0.10 ± 0.05	0.0585	0.08	0.12	40.2
	**Vegetation cover (%)**	**0.12** **± 0.05**	**0.0177**	**0.13**	**0.13**	**38.3**
	**Shrub cover (%)**	**−0.17** **± 0.05**	**0.0004**	**0.21**	**0.31**	**33.2**
	Litter cover (%)	−0.01 ± 0.05	0.9560	0.01	0.01	43.3

## Data Availability

The data presented in this study are available on request from the corresponding author.

## Supplementary Materials

The following material is available online at https://www.mdpi.com/article/10.3390/insects14010083/s1, Table S1: Abundances of all sampled Orthoptera sorted by the family for vineyard types and compartments of vertically oriented vineyards, compartments of terraced vineyards, and canopy. Individuals that were captured in the canopy were not included in the analysis. The status of conservation concern is given according to the Red List for Orthoptera in Germany (Maas et al. 2011) [41], with endangered species highlighted in gray. Table S2: Effects of a local vineyard and landscape parameters on Caelifera and Ensifera species richness and density and density of the two most abundant species, *Chorthippus biguttulus,* and *Oedipoda caerulescens* analyzed using vineyard types. The presented estimates and *p*-values are obtained from the single models Y ~ Intercept + Predictor with ‘vineyard triplet’ as a random factor. R^2^ marginal gives explained variation without and R^2^ conditional with the random factor. Significant predictors (*p* < 0.05) are printed in bold; SNH: semi-natural habitat. Table S3: Effects of a local vineyard and landscape parameters on Orthoptera, Caelifera, and Ensifera species richness and density and density of the two most abundant species, *Chorthippus biguttulus,* and *Oedipoda caerulescens* analyzed using vineyard compartments. The presented estimates and *p*-values are obtained from the single models Y ~ Intercept + Predictor with ‘vineyard row’ as a random factor. R^2^ marginal gives explained variation without and R^2^ conditional with the random factor. Significant predictors (*p* < 0.05) are printed in bold; SNH: semi-natural habitat. Table S4: Correlation of environmental and landscape variables with the first two DCA components related to Orthoptera species composition in vineyard compartments. Variables that were significantly related to the DCA axes (*p* < 0.05, 9999 permutations) are printed in bold. Asterisks indicate significance levels (*p* < 0.05 = *, *p* < 0.005 = **, *p* < 0.001 = ***); SNH: semi-natural habitat. Table S5: Correlation of environmental and landscape variables with the first two PCA components related to Orthoptera species composition in vineyard types. Variables that were significantly related to the PCA axes (*p* < 0.05, 9999 permutations) are printed in bold. Asterisks indicate significance levels (*p* < 0.05 = *, *p* < 0.005 = **, *p* < 0.001 = ***); SNH: semi-natural habitat. Figure S1: PCA results for Orthoptera species composition of vineyard types. The first component explained 38.99% of the variance, the second component was 22.28%. Dots show sample scores for vertically oriented (red), terraced vineyards (blue), and fallows (green). Environmental and landscape variables that were significantly related to the PCA axes (*p* < 0.05, 9999 permutations) were included post-hoc by projection. Indicator species of vertically oriented vineyards are colored in red and in blue for terraced vineyards. For black-colored species names, no assigned indicator values were available. Cho.big: *Chorthippus biguttulus*; Cho.bru: *Chorthippus brunneus*; Cho.vag: *Chorthippus vagans*; Oec.pel: *Oecanthus pellucens*; Oed.cae: *Oedipoda caerulescens*; Pha.fal: *Phaneroptera falcata*; Pla.alb: *Platycleis albopunctata*.
