# Supplementary material for: Orthopteran Diversity in Steep Slope Vineyards: The Role of Vineyard Type and Vegetation Management
Source: Insects. 2023 Jan 13;14(1):83. doi: 10.3390/insects14010083 (PMC9867116; doi:10.3390/insects14010083)
Supplement: Supplementary file 1 [file insects-14-00083-s001.zip › Supplementary material Table S1.pdf]

Supplementary material S1

Table S1: Abundances of all sampled Orthoptera sorted by family for vineyard types and compartments of vertically oriented vineyards, compartments of terraced vineyards, and canopy. Individuals that were captured in the canopy were not included in the analysis. The status of conservation concern is given according to the Red List for Orthoptera in Germany (Maas et al 2011), with endangered species highlighted in gray. The bold improves the readability of the table.

		Vertically oriented				Terrace				Fallow		
Species	Code	Vegetated row	Open row	Canopy	Total	Embankment	Inter-Row	Canopy	Total		**Total**	Status[1]
**Gomphocerinae**												
Chorthippus biguttulus	Cho.big	58	29	0	**87**	10	15	0	**25**	0	**112**	
Chorthippus brunneus	Cho.bru	6	5	0	**11**	1	6	0	**7**	0	**18**	
Chorthippus dorsatus	Cho.dor	0	0	0	**0**	1	0	0	**1**	0	**1**	
Chorthippus vagans	Cho.vag	2	2	0	**4**	3	0	0	**3**	0	**7**	3
Pseudochorthippus parallelus	Pse.par	1	0	0	**1**	0	0	0	**0**	0	**1**	
**Gryllidae**												
Nemobius sylvestris	Nem.syl	0	0	0	**0**	1	0	0	**1**	0	**1**	
**Meconematinae**												
Meconema meridionale	Mec.mer	0	0	0	**0**	1	0	0	**1**	1	**3**	
**Oecanthinae**												
Oecanthus pellucens	Oec.pel	0	1	1	**2**	13	6	0	**19**	3	**24**	
**Oedipodinae**												
Oedipoda caerulescens	Oed.cae	1	13	0	**14**	3	15	1	**19**	0	**32**	V
Oedipoda germanica	Oed.ger	2	1	0	**3**	0	0	0	**0**	0	**3**	1
**Phaneropterinae**												
Phaneroptera falcata	Pha.fal	0	0	0	**0**	3	2	0	**5**	0	**5**	
Phaneroptera nana	Pha.nan	0	0	0	**0**	0	0	0	**0**	1	**2**	
**Tettigoniinae**												
Leptophyes punctatissima	Lep.pun	0	0	1	**1**	0	0	0	**0**	1	**2**	
Pholidoptera griseoaptera	Pho.gri	0	0	0	**0**	1	0	0	**1**	0	**1**	
Platycleis albopunctata	Pla.alb	4	4	0	**8**	14	1	0	**15**	0	**23**	
**Total**		**74**	**55**	**2**	**131**	**51**	**45**	**1**	**97**	**6**	**234**	

[1] Status of conservation concern with V – near threatened, 3 – endangered, 1 – threatened with extinction
