# Supplementary material for: Orthopteran Diversity in Steep Slope Vineyards: The Role of Vineyard Type and Vegetation Management
Source: Insects. 2023 Jan 13;14(1):83. doi: 10.3390/insects14010083 (PMC9867116; doi:10.3390/insects14010083)
Supplement: Supplementary file 1 [file insects-14-00083-s001.zip › Supplementary material Tables S2-S5.pdf]

Supplementary material S2 – S5

Table S2: Effects of local vineyard and landscape parameters on Caelifera and Ensifera species richness and density, and density of the two most abundant species *Chorthippus biguttulus* and *Oedipoda caerulescens* analysed using vineyard types. The presented estimates and p-values are obtained from the single models Y ~ Intercept + Predictor with 'vineyard triplet' as random factor. R^2 marginal gives explained variation without and R^2 conditional with the random factor. Significant predictors ($p < 0.05$) are printed in bold; SNH: semi-natural habitat.

Dependent variable	Predictor	Estimate ± SE	p-Value	R^2_{marg}	R^2_{cond}	AICc
Caelifera species richness	SNH (%)	0.01 ± 0.14	0.7890	0.01	0.01	140.0
	Vineyard (%)	−0.18 ± 0.14	0.1900	0.03	0.03	138.9
	Forest (%)	0.14 ± 0.14	0.3310	0.02	0.02	139.7
	SNH distance (m)	0.11 ± 0.14	0.4180	0.01	0.00	140.0
	Forest distance (m)	−0.19 ± 0.16	0.2350	0.03	0.00	139.1
	**Vegetation height (cm)**	**−1.15 ± 0.32**	**0.0003**	**0.66**	**0.66**	**116.2**
	**Bare ground cover (%)**	**0.38 ± 0.12**	**0.0016**	**0.14**	**0.14**	**131.5**
	Vegetation cover (%)	0.19 ± 0.12	0.1320	0.03	0.03	138.5
	**Shrub cover (%)**	**−1.21 ± 0.32**	**0.0002**	**0.70**	**0.70**	112.3
	Litter cover (%)	0.06 ± 0.14	0.6766	0.01	0.01	140.4
Caelifera density	SNH (%)	0.04 ± 0.05	0.3750	0.02	0.02	33.9
	Vineyard (%)	−0.01 ± 0.05	0.9710	0.01	0.01	34.7
	Forest (%)	0.01 ± 0.05	0.9610	0.01	0.01	34.7
	SNH distance (m)	0.03 ± 0.05	0.4910	0.01	0.00	34.2
	Forest distance (m)	−0.03 ± 0.05	0.5810	0.01	0.00	34.4
	**Vegetation height (cm)**	**−0.14 ± 0.05**	**0.0051**	**0.16**	**0.22**	27.7
	Bare ground cover (%)	0.11 ± 0.05	**0.0352**	0.11	0.11	30.5
	**Vegetation cover (%)**	**0.11 ± 0.05**	**0.0245**	**0.10**	**0.10**	29.9
	**Shrub cover (%)**	**−0.14 ± 0.04**	**0.0013**	**0.19**	**0.21**	25.3
	Litter cover (%)	−0.02 ± 0.05	0.7490	0.01	0.01	34.6
Ensifera species richness	SNH (%)	−0.13 ± 0.16	0.1020	0.08	0.08	117.5
	Vineyard (%)	0.31 ± 0.16	0.0543	0.11	0.11	116.5
	Forest (%)	0.01 ± 0.17	0.9590	0.01	0.08	120.1
	**SNH distance (m)**	**0.42 ± 0.14**	**0.0040**	**0.18**	**0.24**	**113.8**
	Forest distance (m)	−0.09 ± 0.18	0.6260	0.01	0.05	121.8
	**Vegetation height (cm)**	**−0.54 ± 0.26**	**0.0352**	**0.26**	**0.33**	113.8
	Bare ground cover (%)	0.22 ± 0.17	0.2100	0.05	0.20	118.5
	Vegetation cover (%)	0.17 ± 0.15	0.2530	0.04	0.05	1118.9
	**Shrub cover (%)**	**−0.51 ± 0.23**	**0.0340**	**0.24**	**0.27**	114.1
	Litter cover (%)	0.10 ± 0.16	0.5240	0.01	0.08	119.7
Ensifera density	**SNH (%)**	**−0.04 ± 0.02**	**0.0304**	**0.10**	**0.10**	**−45.9**
	**Vineyard (%)**	**0.05 ± 0.02**	**0.0112**	**0.13**	**0.13**	**−47.5**
	Forest (%)	−0.02 ± 0.02	0.3970	0.02	0.02	−42.2
	**SNH distance (m)**	**0.06 ± 0.02**	**0.0029**	**0.17**	**0.00**	**−49.5**
	Forest distance (m)	0.01 ± 0.02	0.5510	0.01	0.01	−41.8
	Vegetation height (cm)	−0.02 ± 0.02	0.3810	0.02	0.02	−42.2
	Bare ground cover (%)	−0.02 ± 0.02	0.4090	0.02	0.02	−42.1

	Vegetation cover (%)	0.01 ± 0.02	0.7080	0.01	0.01	−41.6
	Shrub cover (%)	−0.01 ± 0.02	0.8460	0.01	0.01	−41.5
	Litter cover (%)	0.02 ± 0.02	0.3730	0.02	0.02	−42.2
C. biguttulus density	SNH (%)	−0.01 ± 0.04	0.8194	0.01	0.00	18.8
	Vineyard (%)	0.05 ± 0.04	0.2624	0.03	0.00	17.7
	Forest (%)	−0.02 ± 0.04	0.6048	0.01	0.00	18.6
	SNH distance (m)	0.05 ± 0.04	0.2387	0.03	0.00	17.5
	Forest distance (m)	0.02 ± 0.04	0.7877	0.01	0.00	18.6
	**Vegetation height (cm)**	**−0.08 ± 0.04**	**0.0393**	**0.09**	**0.00**	**14.8**
	Bare ground cover (%)	0.03 ± 0.04	0.4824	0.01	0.00	18.4
	**Vegetation cover (%)**	**0.09 ± 0.04**	**0.0192**	**0.11**	**0.00**	**13.7**
	Shrub cover (%)	−0.01 ± 0.02	0.8460	0.01	0.01	41.5
	Litter cover (%)	0.01 ± 0.04	0.8140	0.01	0.00	18.8
O. caerulescens density	**SNH (%)**	**−0.03 ± 0.01**	**0.0220**	**0.11**	**0.00**	**−83.2**
	Vineyard (%)	0.02 ± 0.01	0.1189	0.05	0.00	−80.6
	Forest (%)	−0.01 ± 0.01	0.9068	0.00	0.00	−78.3
	SNH distance (m)	−0.01 ± 0.02	0.7040	0.01	0.00	−78.4
	Forest distance (m)	−0.02 ± 0.02	0.1984	0.04	0.00	−79.9
	Vegetation height (cm)	−0.02 ± 0.01	0.1590	0.04	0.00	−80.2
	Bare ground cover (%)	0.05 ± 0.02	**<0.0001**	0.31	0.00	−94.3
	Vegetation cover (%)	0.01 ± 0.01	0.7396	0.01	0.00	−78.3
	**Shrub cover (%)**	**−0.03 ± 0.01**	**0.0328**	**0.09**	**0.00**	**−82.6**
	Litter cover (%)	−0.03 ± 0.01	0.0591	0.08	0.00	−81.7

Table S3: Effects of local vineyard and landscape parameters on Orthoptera, Caelifera and Ensifera species richness and density, and density of the two most abundant species *Chorthippus biguttulus* and *Oedipoda caerulescens* analysed using vineyard compartments. The presented estimates and p-values are obtained from the single models Y ~ Intercept + Predictor with 'vineyard row' as random factor. R^2 marginal gives explained variation without and R^2 conditional with the random factor. Significant predictors ($p < 0.05$) are printed in bold; SNH: semi-natural habitat.

**Dependent variable**	Predictor	Estimate ± SE	**p−Value**	**R^2_{marg}**	**R^2_{cond}**	**AICc**
Orthoptera species richness	SNH (%)	0.11 ± 0.09	0.1830	0.02	0.07	186.5
	Vineyard (%)	−0.07 ± 0.09	0.4300	0.01	0.07	187.7
	Forest (%)	0.12 ± 0.08	0.1260	0.02	0.07	186.1
	SNH distance (m)	0.07 ± 0.08	0.3730	0.01	0.07	187.5
	Forest distance (m)	−0.14 ± 0.09	0.1160	0.04	0.07	185.7
	**Vegetation height (cm)**	**0.18 ± 0.06**	**0.0048**	**0.06**	**0.10**	**181.4**
	**Bare ground cover (%)**	**−0.21 ± 0.08**	**0.0158**	**0.07**	**0.19**	**182.6**
	Vegetation cover (%)	0.06 ± 0.08	0.4420	0.01	0.01	187.7
	Shrub cover (%)	0.07 ± 0.07	0.3290	0.01	0.09	187.4
	Litter cover (%)	0.16 ± 0.08	0.0567	0.04	0.17	184.77
Orthoptera density	SNH (%)	0.05 ± 0.06	0.4010	0.01	0.11	88.3
	Vineyard (%)	0.04 ± 0.06	0.5370	0.01	0.11	88.6
	Forest (%)	0.06 ± 0.06	0.3620	0.02	0.11	88.2
	SNH distance (m)	0.03 ± 0.07	0.6420	0.01	0.11	88.8
	Forest distance (m)	−0.04 ± 0.07	0.5780	0.01	0.11	88.7

	Vegetation height (cm)	0.11 ± 0.06	0.0710	0.05	0.17	85.8
	**Bare ground cover (%)**	**−0.12 ± 0.06**	**0.0477**	**0.06**	**0.23**	**85.3**
	**Vegetation cover (%)**	**0.15 ± 0.06**	**0.0161**	**0.09**	**0.16**	**83.5**
	Shrub cover (%)	0.02 ± 0.06	0.7040	0.01	0.13	88.9
	Litter cover (%)	0.04 ± 0.06	0.5700	0.01	0.15	88.7
Caelifera species richness	**SNH (%)**	**0.31 ± 0.11**	**0.0024**	**0.18**	**0.00**	**146.5**
	**Vineyard (%)**	**−0.22 ± 0.09**	**0.0133**	**0.10**	**0.00**	**150.1**
	**Forest (%)**	**0.18 ± 0.08**	**0.0161**	**0.02**	**0.00**	**151.0**
	SNH distance (m)	−0.13 ± 0.09	0.1986	0.03	0.00	154.2
	Forest distance (m)	−0.21 ± 0.11	0.0734	0.09	0.00	152.4
	Vegetation height (cm)	0.05 ± 0.09	0.6037	0.01	0.00	155.7
	Bare ground cover (%)	−0.06 ± 0.11	0.5400	0.01	0.03	155.6
	Vegetation cover (%)	0.09 ± 0.09	0.2650	0.02	0.03	154.8
	Shrub cover (%)	−0.01 ± 0.09	0.9190	0.00	0.03	156.0
	Litter cover (%)	0.01 ± 0.09	0.9640	0.00	0.03	156.0
Caelifera density	SNH (%)	0.11 ± 0.06	0.0762	0.06	0.16	77.4
	Vineyard (%)	0.01 ± 0.06	0.9920	0.00	0.16	80.4
	Forest (%)	0.05 ± 0.06	0.4590	0.01	0.16	79.8
	SNH distance (m)	−0.01 ± 0.06	0.9680	0.00	0.16	80.4
	Forest distance (m)	−0.03 ± 0.06	0.5780	0.01	0.16	80.1
	Vegetation height (cm)	0.04 ± 0.06	0.4710	0.01	0.16	79.9
	Bare ground cover (%)	−0.03 ± 0.06	0.5990	0.01	0.17	80.1
	**Vegetation cover (%)**	**0.12 ± 0.06**	**0.0446**	**0.07**	**0.16**	**76.6**
	Shrub cover (%)	0.01 ± 0.06	0.9910	0.00	0.16	80.4
	Litter cover (%)	−0.04 ± 0.06	0.5050	0.01	0.15	79.9
Ensifera species richness	SNH (%)	−0.21 ± 0.18	0.2390	0.04	0.12	127.2
	Vineyard (%)	0.25 ± 0.18	0.1565	0.06	0.12	126.2
	Forest (%)	−0.05 ± 0.19	0.8012	0.01	0.12	128.5
	**SNH distance (m)**	**0.41 ± 0.15**	**0.0043**	**0.19**	**0.00**	**123.4**
	Forest distance (m)	−0.06 ± 0.19	0.7527	0.01	0.26	130.1
	**Vegetation height (cm)**	**0.41 ± 0.13**	**0.0021**	**0.13**	**0.20**	**119.9**
	**Bare ground cover (%)**	**−0.47 ± 0.20**	**0.0198**	**0.16**	**0.26**	**122.4**
	Vegetation cover (%)	−0.02 ± 0.18	0.8931	0.01	0.12	128.5
	Shrub cover (%)	0.17 ± 0.13	0.1888	0.03	0.14	127.1
	**Litter cover (%)**	**0.42 ± 0.18**	**0.0219**	**0.13**	**0.27**	**122.8**
Ensifera density	SNH (%)	−0.03 ± 0.02	0.1670	0.04	0.22	−40.6
	Vineyard (%)	0.03 ± 0.02	0.2090	0.03	0.22	−40.3
	Forest (%)	−0.01 ± 0.02	0.8730	0.01	0.21	−38.8
	**SNH distance (m)**	**0.06 ± 0.02**	**0.0088**	**0.12**	**0.22**	**−44.9**
	Forest distance (m)	0.01 ± 0.03	0.5550	0.01	0.21	−39.1
	**Vegetation height (cm)**	**0.08 ± 0.02**	**<0.0001**	**0.24**	**0.60**	**−57.4**
	**Bare ground cover (%)**	**−0.06 ± 0.02**	**0.0035**	**0.11**	**0.39**	**−46.4**
	Vegetation cover (%)	−0.01 ± 0.02	0.7430	0.01	0.22	−38.8

	Shrub cover (%)	0.03 ± 0.02	0.1250	0.04	0.30	−41.0
	**Litter cover (%)**	**0.06 ± 0.02**	**0.0018**	**0.13**	**0.42**	**−47.5**
C. biguttulus density	SNH (%)	0.02 ± 0.06	0.7270	0.01	0.23	63.7
	Vineyard (%)	0.07 ± 0.06	0.2040	0.03	0.23	62.2
	Forest (%)	−0.01 ± 0.06	0.8530	0.01	0.23	63.8
	SNH distance (m)	0.04 ± 0.06	0.5090	0.01	0.23	63.4
	Forest distance (m)	0.02 ± 0.06	0.6890	0.01	0.23	63.6
	Vegetation height (cm)	0.08 ± 0.05	0.0849	0.04	0.31	60.9
	Bare ground cover (%)	−0.08 ± 0.05	0.1010	0.04	0.27	61.2
	**Vegetation cover (%)**	**0.12 ± 0.05**	**0.0142**	**0.11**	**0.26**	**58.1**
	Shrub cover (%)	0.03 ± 0.05	0.5980	0.01	0.26	63.5
	Litter cover (%)	0.01 ± 0.05	0.8460	0.01	0.24	63.8
O. caerulescens density	**SNH (%)**	**−0.05 ± 0.02**	**0.0122**	**0.11**	**0.00**	**−51.1**
	Vineyard (%)	−0.03 ± 0.02	0.1282	0.04	0.00	−47.4
	Forest (%)	0.03 ± 0.02	0.1317	0.04	0.00	−47.4
	SNH distance (m)	−0.02 ± 0.02	0.3816	0.01	0.00	−45.9
	Forest distance (m)	−0.03 ± 0.02	0.1791	0.03	0.00	−47.0
	Vegetation height (cm)	−0.02 ± 0.02	0.3462	0.02	0.10	−46.1
	**Bare ground cover (%)**	**0.06 ± 0.02**	**0.0008**	**0.16**	**0.00**	**−55.4**
	Vegetation cover (%)	−0.02 ± 0.02	0.4278	0.01	0.00	−48.8
	Shrub cover (%)	−0.03 ± 0.02	0.2147	0.03	0.00	−46.7
	**Litter cover (%)**	**−0.05 ± 0.02**	**0.0065**	**0.12**	**0.00**	**−52.2**

Table S4: Correlation of environmental and landscape variables with the first two DCA components related to Orthoptera species composition in vineyard compartments. Variables that were significantly related to the DCA axes ($p < 0.05$, 9999 permutations) are printed in bold. Asterisks indicate significance levels ($p < 0.05$=*, $p < 0.005$=**,); SNH: semi-natural habitat.

Variable	DCA1	DCA2	Pr (>r)
SNH (%)	0.2768	−0.9609	0.0610
**Vineyard (%)**	**−0.6143**	**0.9981**	**0.0230 ***
Forest (%)	−0.0978	−0.9952	0.3890
**SNH distance (m)**	**−0.1455**	**0.8943**	**0.0020 ****
Forest distance (m)	0.4476	0.8942	0.4650
Vegetation height (cm)	−0.1116	0.9938	0.2260
**Bare ground cover (%)**	**−0.8813**	**−0.4727**	**0.0290 ***
Vegetation cover (%)	0.4355	−0.9002	0.4490
Shrub cover (%)	0.4596	0.8879	0.3140
**Litter cover (%)**	**0.6258**	**0.7800**	**0.0260 ***

Table S5: Correlation of environmental and landscape variables with the first two PCA components related to Orthoptera species composition in vineyard types. Variables that were significantly related to the PCA axes ($p < 0.05$, 9999 permutations) are printed in bold. Asterisks indicate significance levels ($p < 0.005$=**); SNH: semi-natural habitat.

Variable	PC1	PC2	Pr (>r)
SNH (%)	0.0842	−0.9965	0.1370
Vineyard (%)	0.4296	0.9030	0.1920
Forest (%)	−0.9833	−0.1823	0.6530
SNH distance (m)	0.8418	0.5398	0.4540
Forest distance (m)	0.4777	0.8785	0.2390
Vegetation height (cm)	−0.5822	−0.8130	0.7890
**Bare ground cover (%)**	**−0.1889**	**−0.9819**	**0.0060** **
Vegetation cover (%)	0.9774	0.2113	0.4920
Shrub cover (%)	−0.7962	0.6051	0.0790
Litter cover (%)	0.1444	0.9896	0.0580

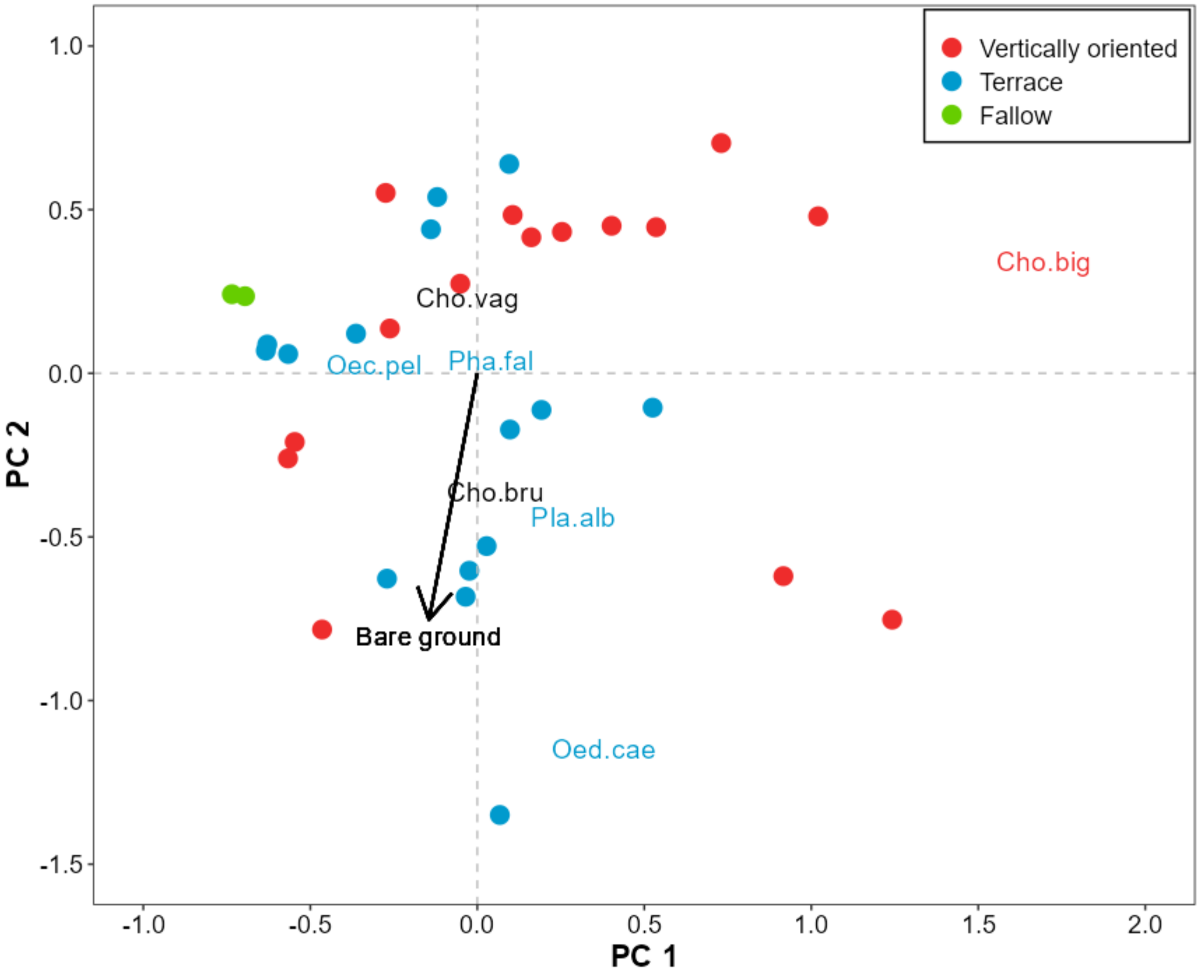

Figure S1. PCA results for Orthoptera species composition of vineyard types. The first component explained 38.99 % of variance, the second component 22.28 %. Dots show sample scores for vertically oriented vineyards (red), terraced vineyards (blue) and fallows (green). Environmental and landscape variables that were significantly related to the PCA axes ($p < 0.05$, 9999 permutations) were included post-hoc by projection. Indicator species are colored in red for vertically oriented vineyards and in blue for terraced vineyards. For black-colored species names, no assigned indicator values were available. Cho.big: *Chorthippus biguttulus*;

Cho.bru: *Chorthippus brunneus;* Cho.vag: *Chorthippus vagans;* Oec.pel: *Oecanthus pellucens*; Oed.cae: *Oedipoda caerulescens;* Pha.fal: *Phaneroptera falcata*; Pla.alb: *Platycleis albopunctata*.
