# Supplementary figures and images for: Orthopteran Diversity in Steep Slope Vineyards: The Role of Vineyard Type and Vegetation Management
Source: Insects. 2023 Jan 13;14(1):83. doi: 10.3390/insects14010083 (PMC9867116; doi:10.3390/insects14010083)

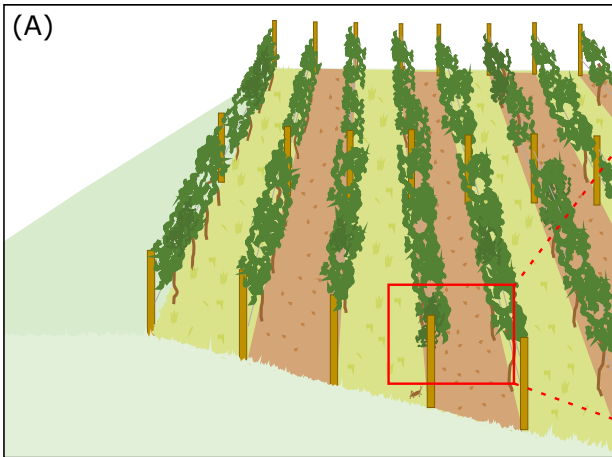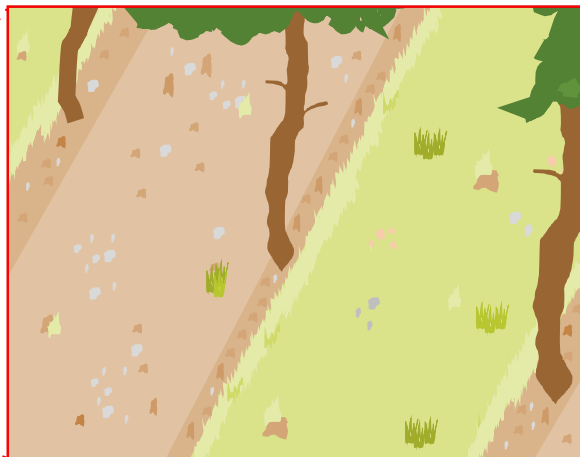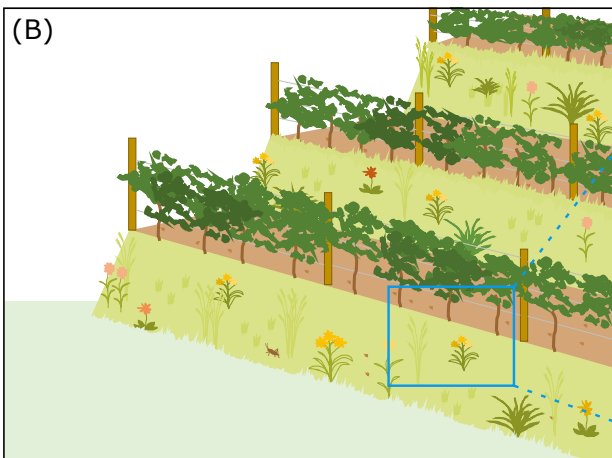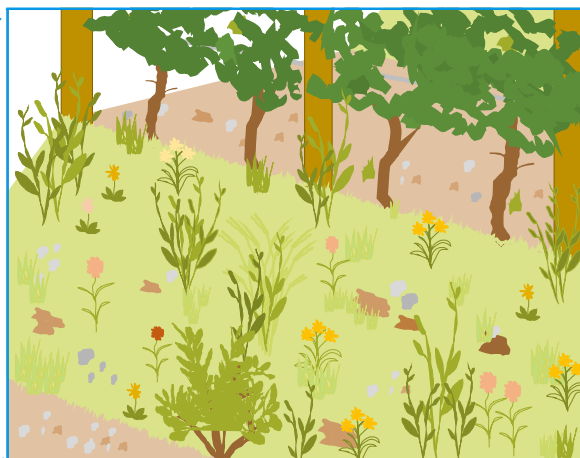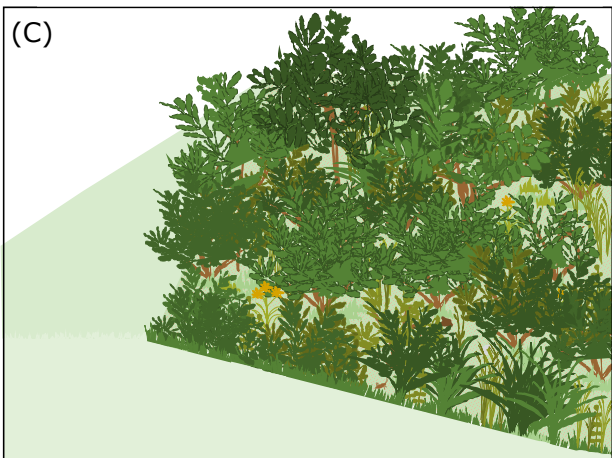

Supplement: Supplementary file 1 [file insects-14-00083-s001.zip › insects-2114301-figures-author proofed.pdf]
